# Effects of Weather and Heliophysical Conditions on Emergency Ambulance Calls for Elevated Arterial Blood Pressure

**DOI:** 10.3390/ijerph120302622

**Published:** 2015-02-27

**Authors:** Jone Vencloviene, Ruta M. Babarskiene, Paulius Dobozinskas, Gintare Sakalyte, Kristina Lopatiene, Nerijus Mikelionis

**Affiliations:** 1 Department of Environmental Sciences, Vytautas Magnus University, Donelaicio St. 58, Kaunas 44248, Lithuania; 2 Department of Cardiology, Lithuanian University of Health Sciences, Eiveniu Str. 2, Kaunas LT-50028, Lithuania; E-Mails: ruta.babarskiene@kaunoklinikos.lt (R.M.B.); gsakalyte@yahoo.com (G.S.); 3 Department of Disaster Medicine, Lithuanian University of Health Sciences, Eiveniu Str. 4, Kaunas LT-50028, Lithuania; E-Mail: paulius@smp.lt; 4 Department of Orthodontics, Lithuanian University of Health Sciences, Luksos-Daumanto Str. 6, Kaunas LT-50106, Lithuania; E-Mail: klopatiene@zebra.lt; 5 Kaunas Emergency Medical Service Station, KaunasLT-51271, Lithuania; E-Mail: mikelionis@greitojipagalba.lt

**Keywords:** emergency ambulance calls, arterial blood pressure, weather, risk

## Abstract

We hypothesized that weather and space weather conditions were associated with the exacerbation of essential hypertension. The study was conducted during 2009–2010 in the city of Kaunas, Lithuania. We analyzed 13,475 cards from emergency ambulance calls (EACs), in which the conditions for the emergency calls were made coded I.10–I.15. The Kaunas Weather Station provided daily records of air temperature (T), wind speed (WS), relative humidity, and barometric pressure (BP). We evaluated the associations between daily weather variables and daily number of EACs by applying a multivariate Poisson regression. Unfavorable heliophysical conditions (two days after the active-stormy geomagnetic field or the days with solar WS > 600 km/s) increased the daily number of elevated arterial blood pressure (EABP) by 12% (RR = 1.12; 95% confidence interval (CI) 1.04–1.21); and WS ≥ 3.5 knots during days of T < 1.5 °C and T ≥ 12.5 °C by 8% (RR = 1.08; CI 1.04–1.12). An increase of T by 10 °C and an elevation of BP two days after by 10 hPa were associated with a decrease in RR by 3%. An additional effect of T was detected during days of T ≥ 17.5 °C only in females. Women and patients with grade III arterial hypertension at the time of the ambulance call were more sensitive to weather conditions. These results may help in the understanding of the population’s sensitivity to different weather conditions.

## 1. Introduction

Arterial hypertension (AH) is one of the main risk factors of cardiovascular diseases and unfavorable prognosis. Patients with elevated arterial blood pressure are frequently found to have cardiac dysfunctions or structural abnormalities (e.g., systolic and diastolic dysfunction or left ventricular hypertrophy), and the progression of the condition leads to coronary artery atherosclerosis, heart failure, and an increased risk of arrhythmias and sudden death [1]. AH is the result of various environmental and genetic factors as well as the expression of those factors in the human body. Together with the aging process, hypertension is the main risk factor contributing to the increase in cardiovascular morbidity and mortality in postmenopausal women, with a prevalence of around 60% in women older than 65 years. Considering that hypertension is a modifiable risk factor, the understanding of its epidemiology and pathophysiology and the development of appropriate therapeutic strategies are conceivably crucial in reducing cardiovascular risk. The high prevalence of hypertension in older women is largely due to the progressive stiffening of the arterial structure which accompanies the aging process in both sexes. However, the abrupt fall in circulating estrogen levels might independently contribute to the rise in blood pressure, through partly unknown mechanisms, such as a direct effect on the arterial wall, the activation of the renin-angiotensin system and of the sympathetic nervous system. Postmenopausal hypertension fosters the development of left ventricular hypertrophy and is the main factor contributing to coronary artery disease, chronic heart failure and stroke in older women [2].

Hypertension is found in approximately 8% of women aged 20 to 44 years; obesity is of particular importance in this population because it affects 93.0% of young women in the United States. It is associated with a greater than fourfold higher risk of hypertension and is potentially modifiable. Premenopausal women are at a higher risk for developing target organ damage (specifically microalbuminuria and left ventricular hypertrophy but are at a lower risk for clinical cardiovascular diseases than men of comparable age [3]. Hypertension severity also increases with age: 48.8% of women aged 60 to 79 years and 63% of women 80 years and older have stage 2 hypertension (BP 160/100 mm Hg) and/or receive antihypertensive therapy. Prevalence approaches 60% in women older than 65 years, largely because of progressive arterial stiffening and abruptly falling estrogen levels, which in turn activate the renin-angiotensin-aldosterone and sympathetic nervous systems [2]. Systolic and pulse pressures are higher in women older than 45 years, whereas diastolic pressures lower in women across all age groups, compared with age-matched men [4].

Over the last five years, a number of studies have been published on the effects of environmental factors on blood pressure [5]. The increase in blood pressure has been associated with short- and long-term exposure to fine particulate matter [6,7,8], traffic noise [9,10,11,12], and cold [13,14,15], as well as with seasonal variation [16,17,18]. Increased geomagnetic activity has also been linked to blood pressure elevation [19,20].

In Kaunas City Emergency Ambulance Service, about 38.4% of emergency ambulance calls (EAC) for cardiovascular diseases were due to elevated arterial blood pressure (EABP; ICD-10 codes I10–I15), and 80% of the patients were females. To improve the quality of work of the emergency medical service, it would be relevant to evaluate the risk of an increase in the number of EABP cases associated with environmental factors, *i.e.*, to identify the complexes of meteorological and heliophysical conditions that are associated with the exacerbation of arterial hypertension. It would also be relevant to evaluate the sensitivity of specific populations (especially women) to weather and space weather conditions. The meteorological, geomagnetic, and other space weather data are easily accessible and predictable, and thus it would be expedient to analyze their combined effect on the increase in arterial blood pressure (ABP) and the exacerbation of arterial hypertension.

The aim of the study was to assess the relation between short-term variations in the meteorological and space weather conditions and the risk of daily emergency ambulance calls for elevated arterial blood pressure. A better understanding of the relationship between weather conditions and the daily number of EAC for EABP will facilitate the introduction of preventives strategies into practice with respect to weather and space weather conditions.

## 2. Experimental Section

### 2.1. Study Setting and Population

The study was conducted in the city of Kaunas, with a population of 306,000 inhabitants. The staff of the ambulance service in Kaunas consists of 50 physicians, 103 nursing specialists in emergency medical care, 69 paramedics, and 35 ambulance traffic controllers. Usually, the ambulance team consists of a physician and a paramedic. The ambulance vehicle is equipped for providing emergency care. In individual cases, depending on the clinical situation, the ambulance crew may consist of a single paramedic or a specialized medical assistance team. Any citizen irrespective of their place of residence, age, ethnicity or race may call the ambulance by phone free of charge in case of an acute health disorder. The ambulance service operates 24/7. Emergency medical assistance is always provided when needed. Upon receiving the call, the ambulance traffic controller consults the patient prior to the arrival of the ambulance or in special cases, coordinates first aid actions until the arrival of a healthcare specialist. In cases of life-threatening situations, the ambulance arrives within 10–15 min, and in other cases—within 30 min.

This was retrospective study using routinely collected data on emergency ambulance calls and daily weather and space weather variables. The study was conducted from 1 January 2009 to 31 December 2010. The patients had essential hypertension, and were administered antihypertensive medications by their family physicians. They had a possibility to monitor their blood pressure and to evaluate the efficiency of the treatment at home. Such patients usually fill out their arterial blood pressure monitoring diary where they indicate their arterial blood pressure (ABP). Ambulance calls were received from patients who against the background of their usual antihypertensive pharmacological treatment suddenly experienced a rise in arterial blood pressure by more than 20 mmHg and additional clinical symptoms such as chest pain, headache, dizziness, or other unusual symptoms. Upon arrival, an ambulance crew member filled out a special clinical evaluation form (form No. 110/a) where he or she recorded the main complaint, the anamnesis, the findings of the clinical examination (heart rate, ABP, clinical signs of heart failure, and ECG parameters), the prescribed home treatment, and the coding of the diagnosis according to the international classification of diseases (ICD-10). We reviewed these forms, and selected patients whose clinical situation was evaluated by the ambulance crew as exacerbation of essential hypertension accompanied by a significant elevation of arterial blood pressure (International Classification of Diseases, 10th Revision (ICD-10)—I.10–I.15).

During this period, we analyzed 13,475 such emergency calls cards (form No. 110/a) from Kaunas city ambulance service. No personal data of the patients were used for the analysis. Elevated arterial blood pressure was evaluated according to the ESH/ESC 2013 Guidelines for the Management of Arterial Hypertension [1]: grade I hypertension—systolic ABP of 140–159 mmHg and/or diastolic ABP of 90–99 mmHg, grade II—systolic and diastolic ABP of 160–179 and/or 100–109 mmHg, respectively, and grade III hypertension—systolic and diastolic ABP ≥ 180 and/or ≥110 mmHg, accordingly. We used the most important data from the cards: age, sex, ABP and heart rate at the time of the ambulance call, and whether first aid was provided at home or the patient was transferred to the hospital. We analyzed the pattern of associations between daily weather and space weather conditions and the daily number of EAC for EABP. The analysis was conducted in men and women—both older (>65 years) and younger, in patients with grade III ABP detected at the time of the ambulance call, as well as in those with lower ABP levels (grade I–II).

### 2.2. Weather and Space Weather Data

The National Environmental Department and the Kaunas Weather Station provided daily records of weather for the study period. Data were available for the following variables: minimal, maximal, and mean daily air temperature (T) (°C), wind speed (WS) (knots), relative humidity (RH) (%), and barometric pressure (BP) (hPA). We also used day length (hours)—the period between sunrise and sunset. Because literature data suggests the influence of air temperature on ABP, we also evaluated the effect of other factors at four different air temperature intervals whose margins were set depending on the number of EAC changes during the 24-hour period.

The Ap index, proton temperature, proton density, and solar wind speed were used as space weather variables. The daily space weather data were downloaded from the National Geophysical Data Center’s OMNIWeb data base (http://omniweb.gsfc.nasa.gov/). In the analysis, the geomagnetic activity was classified as quiet (Ap < 8), unsettled (8 ≤ Ap < 16), and active-stormy (Ap ≥ 16). We investigated the lag 0–7 day’s effect of weather variables on the daily number of EAC for EABP.

### 2.3. Data Analysis

The daily number of EAC for EABP by separate patient groups is presented as mean value ± standard error (SE). The effect of the day of the week was analyzed by including the day’s categories in the Poisson regression model. After the analysis of the mean values of the numbers of EAC and the *p*-values of the regression coefficients in the Poisson regression model, we grouped the days of the week in successive categories: Monday–Tuesday, Wednesday–Sunday, national holidays, and other holidays, not coinciding with weekends. To compare the average daily number of calls on the days of cold and warm periods, we used a t-test. To detect the short-term effect of the environmental conditions, Spearman’s correlation was applied to assess the linear relation between the daily number of EAC and continuous weather variables; also, we used Poisson regression, as the daily numbers of EAC is a count variable. In the analysis, the variables T, day length, WS, RH, and BP were also used as continuous and categorical. A graphical analysis was used to identify the intervals of the weather variable associated with low, moderate, and high number of EAC, or with the U-shaped association. The cumulative effect of two qualitative variables was investigated by including the interaction term in the Poisson regression model. By using the heliophysical data, the binary variable Helio reflecting the unfavorable daily heliophysical conditions was created. It equaled 1 if the daily solar wind speed was ≥600 km/s or an active-stormy geomagnetic activity level was detected two days after; otherwise, Helio = 0. The complex association between weekdays, day length, weather and Helio variables, and the daily number of EAC for EABP was evaluated by using the multivariate Poisson regression. As factors, the regression model included the days of the week as categorical variable (reference category Wednesdays-Sundays without holydays), day length—as a continuous variable, and weather variables—as categorized or continuous; also, interactions between air temperature categories and other weather variable were used. To assess the impact of environmental variables, we presented the percentage increase and the rate ratio (RR) in the daily number of EAC, 95% confidence interval, and *p*-values of coefficients in the Poisson regression. Statistical analysis was performed using SPSS 19 software.

## 3. Results and Discussion

### 3.1. Results

#### 3.1.1. The Effect of Air Temperature, Seasonality, and Day of the Week

There were 13,475 emergency calls for EABP during the 730 days of the study—on average, 19 ± 0.2 calls per day (ranging from 5 to 41 calls, quartiles: 15, 18, and 22). The patients’ mean age was 67 years, and 50.3% (6783) of the patients were 70 years of age and older; 78.5% (10,576) of the patients were females. Women tended to be older—their mean age was 69 ± 0.2 years, compared to 59.6 ± 0.4 years in males. The frequency of ambulance calls received from female patients increased with the patients’ age (Table 1). Mean arterial blood pressure registered on the arrival of the ambulance was 184/100 mmHg, and mean heart rate—81 beats per minute (bpm). In women, mean systolic ABP was 185 ± 0.3 mmHg, which was significantly higher than that in men (181 ± 0.6 mmHg). Grade III hypertension was found in 66.5% (8959) of cases—in 60.5% of men, and in 68% of women (*p* < 0.001). In 21.7% (2884) of cases, heart rate was ≥90 bpm. The mean values of daily EAC for EABP in patient‘s groups were 4 ± 0.1 for men, 14 ± 0.2—for women, 7 ± 0.1—for younger patients, and 12 ± 0.1—for older patients. The daily mean rate of ambulance calls was 12 ± 0.2 for patients with grade III hypertension, and 6 ± 0.1—for those with grade I-II hypertension.

**Table 1:** The distribution of patients by age and sex.

Age	Male		Female		All	
	N	%	N	%	N	%
<30years	202	7.0	98	0.9	300	2.2
30–39 years	232	8.0	209	2.0	441	3.3
40–49 years	365	12.6	687	6.5	1052	7.8
50–59 years	532	18.3	1420	13.4	1952	14.5
60–69 years	581	20.0	2366	22.4	2947	21.9
70–79 years	645	22.2	3222	30.5	3867	28.7
80–89 years	300	10.3	2336	22.1	2636	19.6
≥90 years	43	1.5	238	2.3	281	2.1

**Figure 1:**
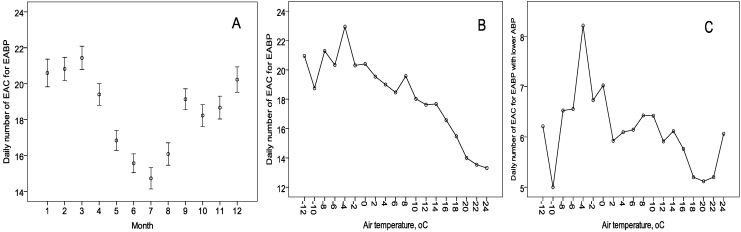
Monthly variation of the mean daily number of emergency ambulance calls for elevated arterial blood pressure (**A**) and the variation of the mean daily number of calls depending on daily air temperature in all patients (**B**) and in patients with grade I-II hypertension at the time of the ambulance (**C**).

There were many more calls during the September–April period (daily mean rate 20 ± 0.2), while during the warmer period—May–August—the mean rate of emergency calls per day was significantly lower—16 ± 0.3 (Figure 1A). The same monthly distribution of ambulance calls for EABP was observed in all patient groups. The daily number of calls was associated with the day of the week: the RRs of the number of ambulance calls were 1.44 (1.23–1.68) on national holiday days, and 1.07 (1.03–1.11)—on Monday-Tuesday, compared to Wednesday–Sunday without national holidays or other holidays not coinciding with weekends; this trend was observed in all patient groups.

The increase in air temperature by 10 °C and the elongation of day length by 1 h was associated with a 10% and 3% decrease (*p* < 0.001) in RR for the daily number for EAC, respectively; the strongest correlation was observed on the days of the calls (lag 0). A J-shaped association between air temperature and the number of EAC was observed; the quadratic term of T was significant in the regression model. Figure 1B shows variations in the mean number of EAC for EABP around the mean value 20.8 in the presence of mean daily T below 1.5 °C, and T ranging between 1.5 and 12.5 °C (mean value—18.8). In case of a higher T, the mean EAC tended to decrease with increasing T in all patient groups – except for those with lower ABP. Considering the daily dynamics of mean EAC rates, the following qualitative classification of air temperature is applicable: T < 1.5 °C (30.1% days); 1.5 ≤ T <1 2.5 °C (35.6% days); 12.5≤ T < 17.5 °C (19.2% days), and T ≥ 17.5 °C (15.1% days).

The strongest negative association between air temperature and rate ratio was detected in patients with grade III AH and in older patients (a decrease in RR by, respectively, 12% and 11% for an increase in T by 10 °C), while in patients with grade I–II AH, the decrease was 6%. The association of the mean daily number of EAC with air temperature in patients with grade I–II AH is presented in Figure 1C.

It shows that more of calls where grade I-II AH was detected occurred when T ranged between −6.5 and +1.5 (°C) (mean value—7 ± 0.3). Such temperatures were mostly registered in January-March (58.3% of days) and during October-December (19.3% of days). The lowest number of ambulance calls per day in case of grade I-II hypertension was registered on days with T ranging between 17.5 and 23.5 (°C) (mean value—5 ± 0.3); 92.6% of days with such T occurred in summer. If the mean air temperature exceeded 23.5 °C, an average of 6 ± 0.9 patients per day were found to have grade I–II hypertension at the time of the emergency ambulance call. However, no statistically significant increase in the risk of such patient was detected, compared to days with lower air temperature.

#### 3.1.2. The Effect of Barometric Pressure, Relative Humidity, Wind Speed, and Heliophysical Conditions

**Figure 2:**
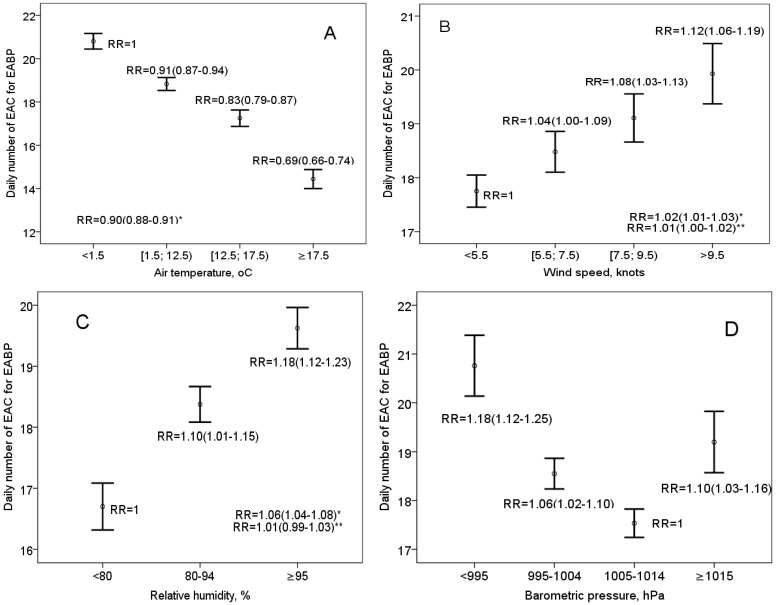
The association between the mean daily value of emergency ambulance calls for elevated arterial blood pressure and daily meteorological variables (* the RR associated with the increase of T by 10 °C, WS by 1knot, and RH by 10%; ** adjusted for T).

In the analysis of univariate associations, significant correlations between the daily number of EAC and other meteorological variables were found. A graphical analysis showed a dose-response relationship between the daily number of EAC for EABP and the daily mean WS and RH, and a U-shape relationship - between EAC and BP (Figure 2). An increase in the RR of the daily number of EAC for EABP was detected in the presence of mean wind speed ≥ 5.5 knots, humid air (RH ≥ 80%), and two days after low (BP < 995 hPa) or high (BP ≥ 1015 hPa) barometric pressure. The effect of day length, low BP, and WS on rate ratio was similar in all patients groups. A 10% increase in RH was associated with a 4%–7% increase in RR in separate patient groups. The negative effect of higher BP was not detected in men, but it was stronger in patients with grade I–II AH.

It is noteworthy that the distribution of BP, RH, and WS categories used in Figure 2 was dependent on air temperature: 54.7% of cases of RH ≥95% occurred when the mean daily temperature was below 1.5 °C, and only 6.2% of cases—when the mean daily temperature was ≥17 °C; also, 54.3% of cases of extremely high barometric pressure occurred when T < 1.5 °C, and only 1.2% of cases—when T ≥ 17 °C. Higher wind speed was also more common on days with lower air temperature. After adjustment for air temperature, significant correlations of EAC with WS and low BP were remained, while no association between RH, and no negative effect of high BP was detected (Figure 2).

The analysis of the associations between the number of calls and WS, RH, and BP in separate patient groups showed that in all patient groups, the RRs of the increase of WS by 1 knot were similar, and no associations between EAC and RH (adjusting for T) were detected. A similar effect of low BP adjusting for T was detected in all patients groups, except for patients with grade I–II AH. In this patient group, the U-shaped relationship between EAC and BP, adjusting for T, was detected: two days after higher (≥1015 hPa) or lower (<995 hPa) barometric pressure, rate ratio was 1.08 (0.98–1.19), compared to moderate BP.

**Figure 3:**
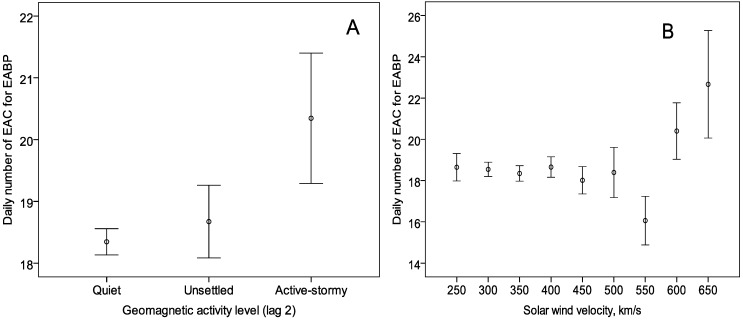
The association between the mean daily values of emergency ambulance calls for elevated arterial blood pressure and daily geomagnetic activity level (lag 2) (**A**) and solar wind speed (**B**).

An increase in the number of EAC for EABP was observed 2 days after an active-stormy geomagnetic activity level and during days with solar wind speed over 600 km/s (Figure 3). The effect of heliophysical conditions was stronger in women and in older patients (Table 2).

**Table 2:** The percentage increase in rate ratio of the daily number of emergency ambulance calls for elevated arterial blood pressure, associated with a change of weather variables in subgroups of patients.

Group	T ^a^	Day length ^b^	BP ^c^ (lag2)	WS ^d^ WS ^†^		RH ^e^ RH ^†^		Helio ^††^
All	−23 *	−5 *	−3 *	2 *	2 *	7 *	12 *	10 *
Men	−16	−1	−4	0	−2	7 *	9 *	−4
Women	−26 *	−6 *	−3 *	3 *	3 *	7 *	13 *	14*
Age ≤ 65 years	−20	−3 *	−3 *	3 *	1	11 *	12 *	7 *
Age > 65 years	−26 *	−6 *	−3 *	1	2 *	4 *	12 *	11 *
3th grade AH	−42 *	−4 *	−4 *	2 *	2 *	7 *	11 *	10 *
1–2th grade AH	19	−5 *	0	3 *	1	7 *	13 *	9

Notes: T—air temperature; WS—wind speed; RH—relative humidity; * *p* < 0.05; ^a^ an increase of T in 10 °C in the interval T ≥ 17.5; ^b^ an increase in the day length by 1hour in the interval 12.5 ≤ T < 17.5; BP ^c^ an increase in barometric pressure by 10 hPa in the interval T < 1.5; ^d^ an increase in WS by 1 knot in the interval 12.5 ≤ T < 17.5; ^e^ an increase in RH by 10% in the interval 12.5 ≤ T < 17.5; ^†^ interaction between air temperature categories: 1.5 ≤ T < 12.5 and (T < 1.5 or T ≥ 12.5); ^††^ the increase on days with solar wind speed ≥ 600 km/s or active-stormy geomagnetic activity level two days after and on other days.

#### 3.1.3. The Multivariate Effects of Environmental Variables

Depending on air temperature, different correlations were observed between the number of calls and WS, BP, RH, and day length (Table 2). During warmer days (T ≥ 17.5 °C), a significant negative association was observed between T and the number of EAC; the decrease in RR was higher in patients with a higher ABP level. Meanwhile, in other air temperature categories, the effect of T was only slight. The negative effect of low BP was stronger during the colder period (T < 1.5 °C) in all patient groups, except for patients with grade I-II AH. A significant effect of day length, WS, and RH was detected only if the mean daily air temperature ranged between 12.5 and 17.5 (Table 2): the effect was similar in all patient groups, except for men. An interaction between air temperature and WS and RH was detected. If the mean daily temperature was lower than 1.5 °C or ≥12.5 °C, a significant increase in the number of calls with increasing WS or RH as observed; conversely, if daily air temperature ranged from 1.5 to 12.5 °C (mostly, in spring and in fall) the mean number of calls tended to decrease with increasing WS or RH. A significant interaction was also found between RH and air temperature categories in all patient groups - only in males it was weaker. A significant interaction was also detected between WS categories and mean daily temperature ranging from 1.5 and 12.5 °C for women and patients with grade I-II AH (Table 2).

The results of multivariate regression models for EAC created for separate patient groups (Table 3) showed that the increase in T by 10 °C was associated with a decrease in RR by 2%–9%, except for patients with lower ABP; the additional effect of T was detected during days with T ≥ 17.5 °C, except for men. The increased BP was associated with a decrease in RR by 3%–5%, except for patients with lower ABP; for these patients, the negative effect of both low and high BP two days after was detected. Only for older patients, the negative effect of lower BP (<995 hPA) was detected. A significant effect of WS was detected in all patient groups, except for men. The effect of the day of the week (Monday–Tuesday) was associated with an increase in RR by 6%–14%, except for patients with grade I–II AH. During national holidays, the RR increased by over 40% in women and in patients with grade III AH. The effect of heliophysical conditions was stronger in older patients. No effect of heliophysical conditions and the effects of T, day length, or WS in separate T categories were detected in men. According the results, presented in Table 3, women and patients with grade III ABP at the time of the ambulance call were more sensitive to weather conditions.

### 3.2. Discussion

According to our results, the mean daily number of EAC for EABP decreased with increasing air temperature; the effect of T was stronger in patients with grade III AH. The associations of air temperature and seasonality with the daily number of EAC for EABP found in our study correspond with the findings of previous studies: lower air temperature was associated with increased blood pressure [5,21,22]; negative associations were found between daily apparent temperature and emergency room visits for hypertension [23]. Lewington *et al*. [15] found that systolic blood pressure was strongly inversely associated with outdoor temperature.

**Table 3:** The multivariate association between environmental variables and the rate ratio (RR (95% CI)) of emergency ambulance calls for elevated arterial blood pressure.

Factors	All patients	Men	Women	Age ≤ 65 years	Age > 65 years	3th grade AH	1–2th grade AH
Monday–Tuesday	1.08 (1.041.12)	1.14 (1.05–1.23)	1.06 (1.01–1.11)	1.12 (1.05–1.19)	1.05 (1.00–1.10)	1.10 (1.05–1.15)	1.03 (0.96–1.10)
National holiday	1.33 (1.13–1.56)	0.98 (0.66–1.47)	1.41 (1.18–1.68)	1.19 (0.91–1.56)	1.40 (1.14–1.70)	1.47 (1.22–1.77)	1.03 (0.75–1.42)
Other holiday *	0.80 (0.68–0.93)	0.66 (0.45–0.95)	0.84 (0.70–1.00)	0.86 (0.67–1.09)	0.75 (0.60–0.92)	0.77 (0.63–0.94)	0.83 (0.64–1.09)
Helio	1.12 (1.04–1.21)		1.16 (1.06–1.26)	1.09 (0.97–1.23)	1.14 (1.03–1.25)	1.12 (1.02–1.23)	1.13 (0.99–1.28)
T (increase in 10 °C)	0.97 (0.94–0.99)	0.91 (0.88–0.95)	0.98 (0.95–1.01)	0.98 (0.94–1.03)	0.96 (0.93–1.00)	0.94 (0.91–0.97)	1.03 (0.98–1.08)
BP (lag2) (increase in 10 hPa)	0.97 (0.95–0.99)	0.95 (0.91–0.99)	0.97 (0.95–1.00)	0.96 (0.93–0.99)		0.96 (0.93–0.98)	
T × T ≥ 17.5)	0.99 (0.98–0.99)		0.99 (0.98–0.99)	0.99 (0.98–0.99)	0.99 (0.98–0.99)	0.99 (0.98–0.99)	0.99 (0.98–1.00)
(Day length–12) × (12.5 ≤ T < 17.5)	0.96 (0.95–0.98)		0.95 (0.94–0.97)	0.97 (0.94–0.99)	0.96 (0.94–0.98)	0.97 (0.95–0.99)	0.95 (0.93–0.98)
(WS ≥ 5.5) × (T < 1.5 or T ≥ 12.5)	1.08 (1.04–1.12)		1.08 (1.04–1.13)	1.09 (1.03–1.15)	1.06 (1.01–1.12)	1.05 (1.00–1.10)	1.12 (1.05–1.20)
(BP < 995) or (BP ≥ 1015) (lag2)							1.08 (1.00–1.16)
WS ≥ 3.5		1.09 (0.98–1.21)					
BP < 995					1.09 (1.02–1.16)		

Notes: * other holidays not coinciding with weekends; T—air temperature, WS—wind speed; BP—barometric pressure; Helio = 1—daily solar wind speed ≥ 600 km/s or active-stormy geomagnetic field was two days after.

The study showed that at daily air temperatures between 1.5 °C and 12.5 °C, the mean daily number of EAC for EABP did not rise with increasing WS or RH. In total, 90.7% of the days with daily air temperature ranging between 1.5 °C and 12.5 °C were in the equinox season; during this period, on 91.7% of days in April, on 88.7% of days in October, and on 81.7% of days in November, air temperature ranged between 1.5 °C and 12.5 °C. It is likely that during these months, the lower WS was associated with worse dilution of pollutants in the atmosphere [24], and a lower RH negatively correlated with particulate matter (PM_10_) levels in Kaunas (http://vddb.library.lt/fedora/get/LT-eLABa-0001:E.02~2010~D_20100614_090215-27623/DS.005.0.01.ETD), which suggests the possibility of blood pressure elevation due to air pollution. Day length significantly reduced the number of EAC for EABP only when the daily air temperature was between 12.5 °C and 17.5 °C; such an air temperature was registered in about 50% of days in May-June and in August, and in about 58% of days in September.

Other researchers in their studies did not find any direct effect of BP, RH, or WS on arterial blood pressure [5,21], but changes in blood pressure were associated with the personal-level environmental temperature (PET) index, calculated by using RH and WS values [5,18]. Our data showed that higher WS (>5.5 knots, except for days with air temperature between 1.5 °C and 12.5 °C) increased the risk ratio of EABP by 1.08-fold; a highly significant positive correlation (r = 0.219, *p* < 0.001) was observed between EAC and WS on warm days (T ≥ 12.5 °C). We will try to explain this fact using data obtained by other researchers.

Yakerson [25] hypothesized that atmospheric electricity is the main influencing climatic factor that could excite multiform biological reactions. During fair weather, an increased wind speed increases air charge density [26] and atmospheric electric field intensity [25]. Thus, the disturbed weather increases atmospheric electricity. Areas of air turbulence often generate extremely low frequency (<300 Hz) electromagnetic fields [27,28]. Currents in the air create extremely low frequency electromagnetic waves as well as 1–3 Hz frequency fluctuations [29]. Studies performed in Siberia have shown that magnetic field fluctuations in the range of 2 Hz increased with increasing WS, at lower and changing BP, and at higher RH [30]. It is likely that in the presence of stronger wind and higher relative humidity, low-frequency electric currents and atmospheric pressure fluctuations negatively affect human physiological state and increase blood pressure, thus increasing the risk of the need for emergency medical assistance. In hypertensive patients, higher blood pressure values were observed on cyclonic days: an increase in blood pressure followed a sudden day-to-day change of the weather pattern going from anti-cyclonic to cyclonic days [31].

According to our data, a U-shaped relation between daily atmospheric pressure and EABP was detected in patients with lower ABP; low BP was associated with a higher risk for calls in the multivariate regression model. Other authors also stated a negative effect of lower BP and BP reduction on human health. Low BP (4-day time lag) increased daily emergency attendance in Hong Kong, adjusting for other weather variables [32]. A V-form relation between daily atmospheric pressure and cardiovascular events rate was detected [33]. A negative effect of low BP on blood pressure levels was reported in hypertensive patients who did not respond to treatment [34].

Unfavourable space weather conditions were also included as a predictor for the number of EAC for EABP. Studies have shown that geomagnetic changes had a statistically significant influence on arterial blood pressure [19,20]. The high-speed solar wind triggers magnetic and ionospheres storms, which may affect the fluctuations in atmospheric pressure and other weather conditions and this, in turn, affects human health.

The analysis of the effect of meteorological and heliophysical variables in separate patient groups showed that air temperature was more associated with a higher (≥180/110 mmHg) blood pressure. Women were more sensitive to changes in space weather conditions and in meteorological conditions during different air temperature intervals. In women, a stronger negative effect was exerted by high WS during days of air temperature changes exceeding the range of 1.5–12.5 °C, and also by unfourable heliophysical conditions. In their analysis of the influence of daily changes in geomagnetic activity on systolic and diastolic blood pressure, Dimitrova and Stoilova [35] established that females were more sensitive to GMA disturbances compared to males, which corresponds to our results. Increased geomagnetic activity affects sympathetic nervous system, and the sympathetic nervous system is predominant in women. The potential for sex to modulate the integrative neural control of the cardiovascular system is beginning to emerge. Greater age-related increases in sympathetic activity and arterial pressure have been documented in women [36]. Recent evidence also suggests that sympathetic activation is linked to the menstrual cycle, estrogen being sympathoinhibitory and progesterone being sympathoexcitatory [37]. Indeed, estrogen receptors, specifically estrogen receptor β, in the paraventricular nucleus and rostroventrolateral medulla have been recently shown to mediate the protective actions of estrogen to attenuate aldosterone/high salt–induced hypertension in mice by blunting sympathetic activation [38]. In young men, muscle sympathetic nerve activity is correlated with total peripheral resistance and inversely related to cardiac output. These relationships do not exist in young women, demonstrating fundamental sex-related differences in the mechanisms regulating blood pressure [39]. There exist sex-related differences in sympathetic responsiveness at the neuromuscular junction. Vasoconstrictor responses to adrenergic nerve stimulation are higher in males than in females, with the differences resolved with ovariectomy [40]. Thus, the decline in the influence of the sex hormones on neurovascular constriction with aging will facilitate increased responsiveness of the vasculature to sympathetic activity. Understanding the interaction of sympathetic outflow with sex hormones may shed light on the causes behind the surge in hypertension in women after menopause [41]. Our data showed that the correlation between air temperature and the daily number of EAC was significantly lower in women than in men. Other researchers did not detect any significant difference between the effect of air temperature in women and in men [42], yet it is likely that women more sensitively reacted to temperature changes than men did, which was reflected in the dynamics of the number of ambulance calls.

According to our data, individuals over 65 years of age were more sensitive to heliophysical disturbances. This may be due to the fact that there were more females among older patients. Older people were also more sensitive to low atmospheric pressure, were somewhat more sensitive to changes in air temperature, and less sensitive—to changes in wind speed. Other researchers have also stated a greater effect of meteorological conditions on older patients. A stronger influence of air temperature and atmospheric pressure on the rate of cardiovascular events and on the number of daily emergency calls was observed in this population [32,33]. It is noteworthy that when analyzing groups of patients younger than 70 years of age, we found no differences in their sensitivity to meteorological conditions.

Other authors in their studies used the day of the week and air temperature to predict the daily number of visits to the emergency department or the daily number of emergency calls [32,43]. Most of the models used to predict the number of patients in need of emergency medical assistance were regression models including calendar variables or times series models [43,44]; these models examined the additive effect of the predictors. Our data revealed a multiplicative effect and an interaction between weather variables.

### 3.3. Limitations

Our study is limited in that we had no data on any personal risk factors—e.g., alcohol use or smoking, stress, or co-morbidities. In addition to that, we did not have any data on other environmental factors that might elevate arterial blood pressure—*i.e.*, air pollution and noise levels in the area of residence, the climatic conditions within peoples’ homes (indoor air temperature and air quality), and time spent indoors. In this study we did not evaluate the effectiveness of pharmacological treatment. All these factors may be seen as confounding factors. Besides, this study lasted only two years, which is not a long period when analyzing changes in seasonality and meteorological factors and their interrelation. For this reason, the evaluation of significant differences and the influence of meteorological factors in the studied subpopulations were complicated.

## 4. Conclusions

According to our results, weather and space weather condition affected the daily number of EAC for EABP, and the effects of wind speed and day length are dependent on air temperature. Unfavorable heliophysical conditions as well as active-stormy geomagnetic activity two days after along with high solar wind speed increased the number of EABP as well. Women and patients with grade III ABP at the time of the ambulance call were more sensitive to weather conditions. These results may help in the understanding of the population‘s sensitivity under different weather conditions. With the knowledge of the weather and space weather forecast, the work of the emergency medical service may be organized accordingly.
